# Immediate postoperative parenteral anticoagulant therapy in patients with mesenteric ischemia after intestinal resection: a retrospective cohort study at a single institute

**DOI:** 10.1186/s12876-023-02691-w

**Published:** 2023-03-08

**Authors:** Hsiao-Tien Liu, Chia-Yu Lai, Jian-Jhou Liao, Yi-Ju Chen, Shao-Bin Cheng, Cheng-Chung Wu

**Affiliations:** 1 Department of Surgery, Taichung Veterans General Hospital, 1650 Taiwan Boulevard Sect. 4, Taichung, 40705 Taiwan, ROC; 2 Organ Transplantation Center, Taichung Tzu Chi Hospital, Buddhist Tzu Chi Medical Foundation, Taichung, Taiwan; 3 School of Medicine, Chung Shan Medical University, Taichung, Taiwan

**Keywords:** Mesenteric ischemia, Parenteral anticoagulant, Intestine resection

## Abstract

**Background:**

Bowel gangrene represents a major fatal event in acute mesenteric ischemia. Intestinal resection is inevitable in patients with peritonitis and bowel gangrene. This retrospective study aimed to elucidate the benefit of postoperative parenteral anticoagulation in patients with intestinal resection.

**Methods:**

Patients with acute mesenteric ischemia and bowel gangrene were recruited retrospectively between January 2007 and December 2019. All patients underwent bowel resection. They were categorized into two groups: patients without immediate parenteral anticoagulant therapy (Group A) and those with immediate parenteral anticoagulant therapy (Group B). Thirty-day mortality and survival were analyzed.

**Results:**

A total of 85 patients were included, with 29 patients in Group A and 56 patients in Group B. Patients in Group B had lower 30-day mortality (16.1%) and a higher 2-year survival rate (45.4%) than patients in Group A (30-day mortality: 51.7%, p = 0.001; 2-year survival rate: 19.0%, p = 0.001). In the 30-day mortality multivariate analysis, patients in Group B had a better outcome (odds ratio = 0.080, 95% confidence interval between 0.011 and 0.605, p = 0.014). Patients in Group B also had a better outcome in the survival multivariate analysis (hazard ratio: 0.435, 95% confidence interval between 0.213 and 0.887, p = 0.022).

**Conclusions:**

Immediate postoperative parenteral anticoagulant therapy improves prognosis in patients with acute mesenteric ischemia treated by intestinal resection.

*Trial registration* This research was retrospectively approved by the Institutional Review Board (IRB) I&II of Taichung Veterans General Hospital (TCVGH-IRB No.CE21256B) on July 28th, 2021. The informed consent waiver was also approved by IRB I&II of Taichung Veterans General Hospital. The Declaration of Helsinki and ICH-GCP guidelines were followed during this study.

## Background

Mesenteric ischemia is characterized by insufficient splanchnic perfusion leading to failure to meet the intestinal metabolic demands, eventually resulting in ischemic tissue injury [1]. Mesenteric ischemia is divided into acute and chronic patterns. There are four different etiological forms of acute mesenteric ischemia (AMI): arterial embolism (EAMI), arterial thrombosis (TAMI), nonocclusive mesenteric ischemia (NOMI), and venous thrombosis (VAMI) [2]. The incidence of AMI is estimated to be approximately 1:1000 of acute hospital admissions in Europe and the USA [3]. This figure accounts for approximately 1:10,000 in Japan, where the incidence of vascular disease is also lower [4]. In the 1990s, the mortality rate was quite high, with an average of 71%, ranging between 59 and 93%. Delayed diagnosis and the existence of intestinal gangrene were also shown to be poor prognostic factors [5].

In the past two decades, early diagnosis with successful revascularization has been able to improve survival, with 30-day mortality rates between 6.9 and 29.6% [6–8]. However, the survival rate remains poor in patients with AMI who have intestinal gangrene and resection treatment, with mortality rates between 27 and 59% [9–12]. Immediate anticoagulation for a patient with VAMI is indicated regardless of whether the patient requires surgical intervention. Heparinization is indicated in patients with EAMI, TAMI, and NOMI, but the period and dosage are controversial [5]. In patients with arterial occlusion, unfractionated heparin and low-molecular-weight heparins (LMWHs) are suggested during the critical perioperative period after revascularization and/or bowel resection [13, 14]. In our institute, subcutaneous enoxaparin is more frequently used than intravenous heparin. To our knowledge, there are few studies on postoperative anticoagulation in patients with AMI and intestinal resection. Therefore, we conducted a retrospective study to elucidate the influence of postoperative anticoagulant use on survival in patients with AMI after emergent intestinal resection.

## Methods

### Patient recruitment

Between January 2007 and December 2019, we retrospectively reviewed patients who underwent emergent small bowel resection for nonmalignant lesions, by operative notes as well as by pathology results. We aimed to identify patients with AMI and intestinal gangrene. Patients who experienced intestinal gangrene and resection were included. The preoperative diagnosis of AMI was determined by contrast computed tomography (CT) scan. Gangrene of the bowel was confirmed by pathological findings. Patients without intestinal resection, intestinal gangrene, or preoperative contrast CT scans were all excluded. Patients with isolated ischemic colitis were also excluded. Patients with intestinal gangrene resulting from adhesion, abdominal wall hernia, internal herniation, or concurrent active malignancy were all excluded.

### Data collection

Preoperative factors were collected, including age, sex, recent major cardiovascular procedure (within 3 months), coronary artery disease, cerebral vascular events, chronic lung disease, essential hypertension, dyslipidemia, liver cirrhosis, atrial fibrillation, type 2 diabetes mellitus, end-stage renal disease with dialysis (both hemodialysis and peritoneal dialysis), and regular use of antiplatelet or anticoagulant agents. The major cardiovascular procedures included coronary arterial bypass, coronary arterial angioplasty/stenting, cardiac valvular surgery, aortic surgery, and peripheral arterial surgery. Preoperative blood cell counts included white cell counts, differential counts (immature band form white cell) [15], platelet counts, the neutrophil-to-lymphocyte ratio (NLR) [16], and hemoglobin levels. Preoperative blood biochemistry results included serum levels of albumin, alanine aminotransferase (ALT), bilirubin, and creatinine. The coagulation test included the prothrombin time (PT) and was expressed by the international normalized ratio (INR). Preoperative shock status was defined as the requirement for vasopressors or inotropes. The types of AMI were determined by preoperative contrast CT scans.

### Prognosis analysis

Patients underwent emergent laparotomy under general anesthesia. The periods between definite diagnosis (by contrast CT scan) and surgery were measured. Operative factors included combined colon resection, end-enterostomy or reanastomosis, length of functional residual small intestine (length between the Treitz ligament and the end-enterostomy or ileocecal area), and blood transfusion. Both postoperative anticoagulant agents and postoperative antiplatelet agents were recorded. Postoperative mortality was defined as death after intestinal resection. The periods between intestinal resection and death were measured. The 30-day mortality was analyzed for short-term results. The prognosis was revealed by the survival curve.

### Statistical method

We used IBM SPSS version 22.0 for the statistical analysis. Continuous data are presented as the median with range. Categorical variables were compared by Pearson’s chi-square or Fisher’s exact as appropriate. Continuous variables were compared by the Mann–Whitney U test (nonparametric method). Multivariate analysis for risk factors for mortality was conducted using a logistic regression model. Prognosis and survival curves were generated by the Kaplan–Meier method. Comparative analyses were conducted by log-rank and Cox regression. Significance was defined as p < 0.05.

## Results

### Overall results

**Fig. 1:**
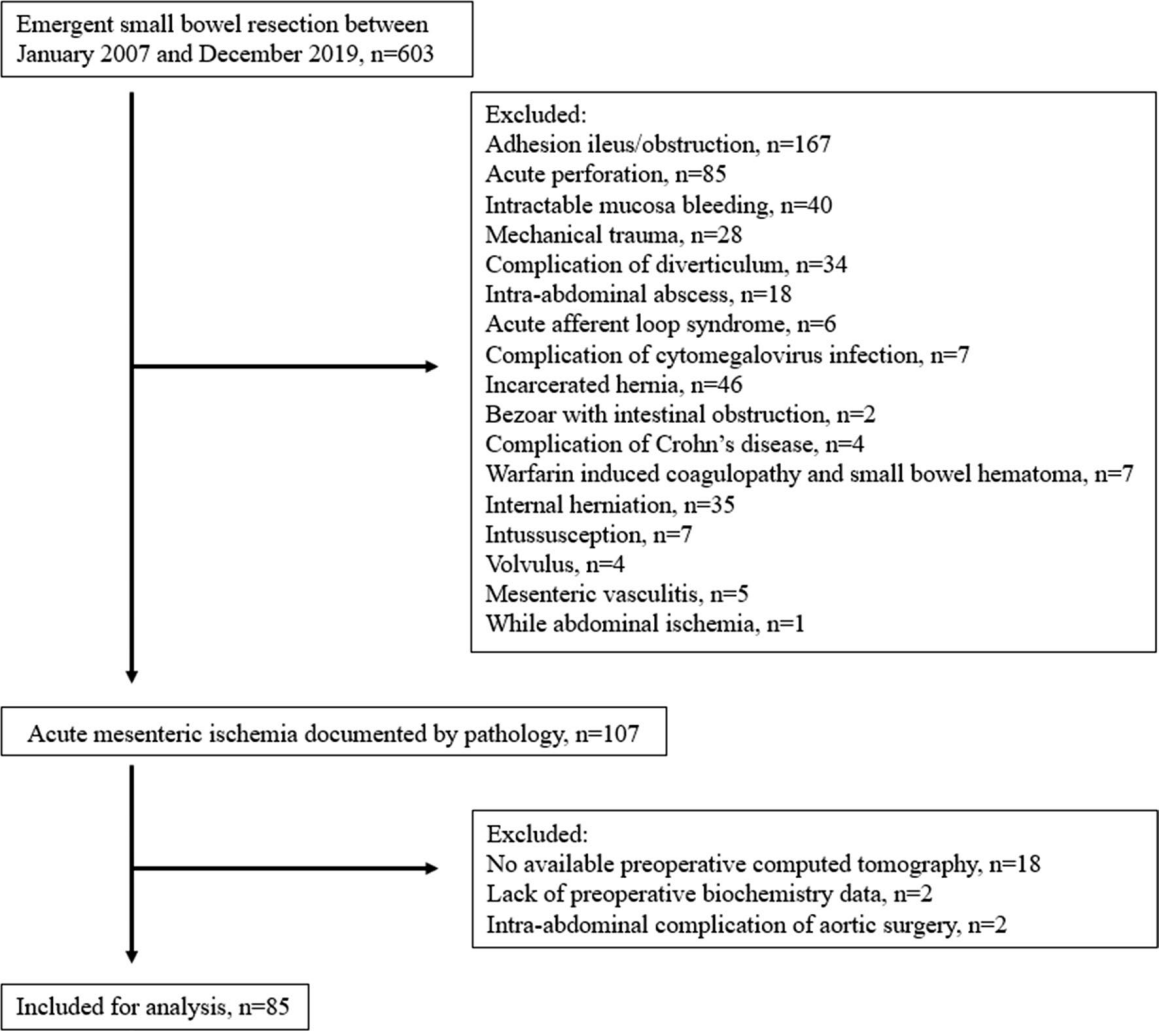
Flow chart

We found six hundred and three patients who underwent emergent small bowel resection for benign lesions between January 2007 and December 2019. One hundred and seven patients with *AMI* were enrolled. Eighteen patients did not have a contrast CT scan before surgery. Two patients experienced complications of aortic surgery. Two patients lacked preoperative biochemistry data. Eventually, a total of 85 patients were included in the analysis (Fig. 1). The median age was 77 years with a range between 23 and 95 years. There were 35 females and 50 males. The numbers of patients with EAMI, TAMI, NOMI, and VAMI were 18, 27, 35, and 5, respectively. The overall 30-day mortality rate was 28.2%. The overall 2-year survival rate was 36.0%. Nineteen patients had recent major cardiovascular procedures. Six patients underwent coronary arterial bypass grafting, 5 patients underwent cardiac valvular surgery, 5 patients underwent aortic surgery, 2 patients underwent peripheral arterial surgery, and 1 patient underwent percutaneous coronary angioplasty with stents.

Of the 85 patients, 40 patients died within 3 months. Definite sepsis (positive bacterial cultures and systemic inflammatory response syndrome) occurred in 21 patients. Among the 19 patients who died of non-septic insults, hepatic failure, noted in 12 patients, was the most common organ failure at death, followed by renal failure and then heart and respiratory failure (Table 1). Ten patients experienced a second abdominal surgery, 4 for abdominal fascia dehiscence, 2 for recurrent intestinal ischemia, 1 for intra-abdominal abscess, 1 for anastomosis leakage, 1 for poor healing of enterostomy, and 1 for second-look operations without any complications.

**Table 1:** Cause of death within 3 months

		Patient number
Sepsis		21
Non-sepsis		19
Organ failure in non-sepsis	Liver	12
	Kidney	7
	Heart	4
	Lung	4

### Postoperative parenteral anticoagulant analysis

The postoperative anticoagulant and antiplatelet drugs included different agents. In our institute, the most commonly used parenteral anticoagulant agent is enoxaparin. We categorized patients into two groups. Patients who did not receive immediate postoperative parenteral anticoagulants were categorized into Group A, and those who had immediate postoperative parenteral anticoagulant treatments were included in Group B. Parenteral anticoagulants were initiated within 24 h after surgery in Group B. There were 29 and 56 patients in Groups A and B, respectively. In Group B, 50 patients had immediate postoperative subcutaneous enoxaparin, 5 patients had intravenous heparin for one day, and then subcutaneous enoxaparin was administered, and 1 patient had intravenous heparin for one day only. The different uses of anticoagulants depended on surgeon’s personal decision and no specific criterion was considered. No patient in Group A received parenteral anticoagulants during hospitalization after surgery. The daily dosages of enoxaparin were between 20 and 120 mg, with one injection given every 24 h or two injections every 12 h. When enteral medication became available, 4 patients received oral antiplatelet agents in Group A. Eighteen patients had oral antiplatelet agents, 12 patients had oral anticoagulants, and 4 patients received both in Group B. In Group B, the interval use of parenteral anticoagulants ranged between 1 and 32 days, with a median of 9 days.

**Fig. 2:**
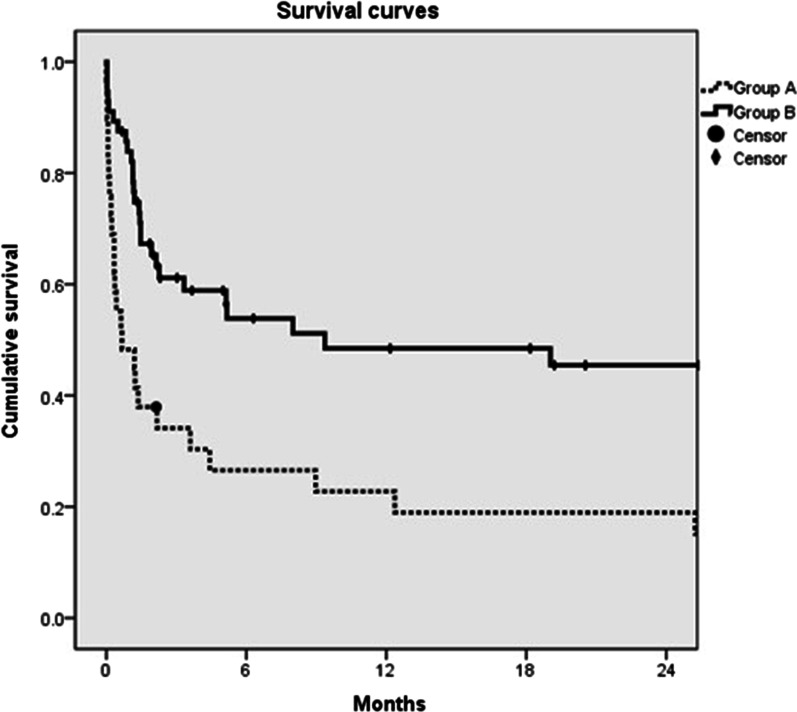
Survival curve. 2-year survival rate. Group A: 19.0%. Group B: 45.4%. p = 0.001

A comparison of demographics and other characteristics between Groups A and B is presented in Table 2. There was no significant difference in background factors between the two groups. The normal range of white blood cells was defined as between 4000 and 12,000/uL [17]. The white blood cell distribution, including total counts and the existence of immature neutrophils, revealed significant differences between the two groups. Patients in Group A had lower levels of serum albumin and hemoglobin, and higher levels of serum creatinine. More patients in Group B underwent combined colon resection and end-enterostomy. Patients had significantly lower 30-day mortality in Group B. The follow-up outcome is revealed by the survival curve in Fig. 2. Patients in Group B showed better median survival periods (2-year survival rates of Groups A and B: 19.0% and 45.4%, respectively, P = 0.001).

**Table 2:** Demographic characteristics of group A and B

	Group A (n = 29)	Group B (n = 56)	P value
*Background factors*			
Age (year)^a^	78 (41–95)	75 (23–92)	0.363
Gender (female/male)	14/15	21/35	0.362
Type (EAMI/TAMI/NOMI/VAMI)	4/10/15/0	14/17/20/5	0.171
*Medical history*			
Recent cardiovascular procedure^b^ (n)	7	12	0.789
Coronary artery disease (n)	9	24	0.352
Cerebral vascular event (n)	5	11	1.000
Chronic lung disease (n)	5	6	0.499
Essential hypertension (n)	18	43	0.204
Dyslipidemia (n)	3	3	0.406
Liver cirrhosis (n)	2	4	1.000
Atrial fibrillation (n)	4	8	1.000
Diabetes mellitus (n)	14	20	0.351
Dialysis (n)	11	10	0.062
Regular antiplatelet or anticoagulant use (n)	10	23	0.642
*Preoperative factors*			
WBC count(/uL)	15,400 (3,250–37,700)	11,685 (890–28,800)	0.031
WBC count out of normal range^c^ (n)	20	28	0.111
Positive band form of WBC (n)	21	26	0.037
NLR (%)	12.7 (2.7–98.0)	13.0 (1.9–97.0)	0.704
Platelet count (/uL)	139,000 (14,000–485,000)	145,500 (21,000–341,000)	0.871
INR of PT	1.17 (0.91–8.77)	1.13 (0.88–3.50)	0.226
Hemoglobin (g/dL)	10.3 (6.1–14.8)	11.9 (6.0–16.6)	0.012
Serum albumin (g/dL)	2.5 (1.2–4.5)	3.1 (1.5–4.5)	0.002
Serum ALT (U/L)	28 (7–3049)	30 (4–1332)	0.714
Serum bilirubin (mg/dL)	0.8 (0.2–6.4)	0.9 (0.3–9.5)	0.451
Serum creatinine (mg/dL)	3.7 (0.9–12.0)	2.5 (0.40–12.1)	0.047
Shock status (n)	14	15	0.057
*Surgical factors*			
Time to surgery (hour)^d^	5.4 (2.14–102.7)	5.5 (1.6–134.0)	0.802
Intraoperative red blood transfusion (n)	2	7	0.712
Combined colon resection (n)	4	22	0.024
End-enterostomy (n)	16	44	0.043
Functional residual small intestine (cm)	170 (5–270)	115 (10–400)	0.820
*Prognosis*			
30-days mortality (n)	15	9	0.001
2-year survival rates (%)	19	45.4	0.001

*EAMI* arterial embolism; *TAMI* arterial thrombosis; *NOMI* non-occlusive mesenteric ischemia; *VAMI* venous thrombosis; WBC: white blood cell; *NLR* neutrophil-to-lymphocyte ratio; INR: international normalized ratio; *PT* prothrombin time; *ALT* alanine aminotransferase

^a^Median (range)

^b^Experiencing major cardiovascular procedure within 90 days before intestine resection

^c^Normal range of WBC: between 4000 and 12,000/uL

^d^Period between definite diagnosis by contrasted computed tomography and surgery

### Prognosis analysis

The risk factors for 30-day mortality were analyzed by univariate and multivariate methods, and the results are shown in Table 3. Patients had a lower mortality rate in Group B (p = 0.001). Recent cardiovascular procedures, preoperative shock status, thrombocytopenia (platelets < 100,000/uL), hypoalbuminemia (serum albumin < 2.8 g/dL), elevated serum ALT (≥ 100 U/L), and elevated serum creatinine (≥ 2.0 mg/dL) had significant impacts on 30-day mortality. After multivariate analysis, thrombocytopenia and elevated serum ALT were significant unfavorable factors. Oral antiplatelets after bowel movement recovery also provided a significant benefit. Patients in Group B still had a better outcome than those in Group A (odds ratio = 0.080, 95% confidence interval between 0.011 and 0.605, p = 0.014).

**Table 3:** 30-day mortality analysis

		Univariate analysis		Multivariate analysis	
		30-days mortality (%)	P value	Odds ratio (95% CI)	P value
Group	A	51.7	0.001	Ref.	0.014
	B	16.1		0.080 (0.011–0.605)	
*Background factors*					
Age	< 65 years	33.3	0.583		
	≥ 65 years	26.6			
Gender	Female	31.4	0.630		
	Male	26.0			
Type	EAMI	22.2	0.292		
	TAMI	25.9			
	NOMI	37.1			
	VAMI	0.0			
*Medical history*					
Recent cardiovascular procedure^a^	No	21.2	0.018	Ref.	0.326
	Yes	52.6		2.765 (0.364–21.028)	
Coronary artery disease	No	30.8	0.624		
	Yes	24.2			
Cerebral vascular event	No	27.5	0.765		
	Yes	31.3			
Chronic lung disease	No	28.4	1.000		
	Yes	27.3			
Essential hypertension	No	20.8	0.428		
	Yes	31.1			
Dyslipidemia	No	29.1	0.671		
	Yes	16.7			
Liver cirrhosis	No	29.1	0.671		
	Yes	16.1			
Atrial fibrillation	No	27.4	0.733		
	Yes	33.3			
Diabetes mellitus	No	21.6	0.139		
	Yes	38.2			
Dialysis	No	26.6	0.583		
	Yes	33.3			
Regular antiplatelet or anticoagulant use (n)	No	23.1	0.221		
	Yes	36.4			
*Preoperative factors*					
WBC count out of normal range^b^	No	24.3	0.628		
	Yes	31.3			
Positive band form of WBC	No	18.4	0.092	Ref.	0.430
	Yes	36.2		0.487 (0.081–2.912)	
NLR	< 23.8	27.0	0.784		
	≥ 23.8	31.8			
Platelet count	≥ 100,000/uL	16.9	0.001	Ref.	0.031
	< 100,000/uL	53.8		10.293 (1.231–86.086)	
INR of PT	≤ 1.7	24.3	0.067	Ref.	0.976
	> 1.7	54.5		0.959 (0.063–14.503)	
Hemoglobin	≥ 12 g/dL	24.2	0.624		
	< 12 g/dL	30.8			
Serum albumin	≥ 2.8 g/dL	16.7	0.008	Ref.	0.406
	< 2.8 g/dL	43.2		2.003 (0.389–10.314)	
Serum ALT	< 100 U/L	17.9	0.000	Ref.	0.029
	≥ 100 U/L	66.7		11.523 (1.283–103.481)	
Serum bilirubin	< 2.0 mg/dL	24.2	0.153		
	≥ 2.0 mg/dL	42.1			
Serum creatinine	< 2.0 mg/dL	11.1	0.020	Ref.	0.716
	≥ 2.0 mg/dL	36.2		1.407 (0.223–8.868)	
Shock status	No	12.5	0.000	Ref.	0.153
	Yes	58.6		3.438 (0.631–18.723)	
*Surgical factors*					
Time to surgery^c^	< 12 h	30.3	0.567		
	≥ 12 h	21.1			
Intraoperative red blood transfusion	No	28.9	1.000		
	Yes	22.2			
Combined colon resection	No	25.4	0.438		
	Yes	34.6			
End-enterostomy	No	16.0	0.122		
	Yes	33.3			
Functional residual small intestine	≥ 100 cm	22.4	0.224		
	< 100 cm	36.1			
*Postoperative medical factor*					
Oral antiplatelets	No	39.0	0.001	Ref.	0.018
	Yes	3.8		0.025 (0.001–0.528)	

*EAMI* arterial embolism; *TAMI* arterial thrombosis; *NOMI* non-occlusive mesenteric ischemia; *VAMI* venous thrombosis; *WBC* white blood cell; *NLR* neutrophil-to-lymphocyte ratio; *INR* international normalized ratio; *PT* prothrombin time; *ALT* alanine aminotransferase; *CI* confidence interval; *Ref.* reference

^a^Experiencing major cardiovascular procedure within 90 days before intestine resection

^b^Normal range of WBC: between 4000 and 12,000/uL

^c^Period between definite diagnosis by contrasted computed tomography and surgery

Table 4 shows all factors influencing survival. To make the analysis feasible, the types of AMI were categorized into nonarterial occlusion (NOMI and VAMI) and arterial occlusion (EAMI and TAMI). Prognosis was affected by abnormalities in white cell counts, thrombocytopenia, coagulopathy, hypoalbuminemia, elevated serum ALT, hyperbilirubinemia, elevated serum creatinine, preoperative shock status, combined colon resection, end-enterostomy, and functional residual small intestine. After multivariate analysis, postoperative parenteral anticoagulant treatments still conferred a survival advantage (hazard ratio: 0.435, with 95% confidence interval between 0.213 and 0.877, p = 0.022).

**Table 4:** Prognosis analysis

		Univariate analysis		Multivariate analysis	
		2-year survival rate (%)	P value	Hazard ratio (95% CI)	P value
Group	A	19.0	0.001	Ref.	0.022
	B	45.4		0.435 (0.213–0.887)	
*Background factors*					
Age	< 65 years	47.6	0.525		
	≥ 65 years	29.9			
Gender	Female	32.3	0.619		
	Male	38.8			
Type	Non-arterial occlusion	47.5	0.366		
	Arterial occlusion	27.5			
*Medical history*					
Recent cardiovascular procedure^a^	No	36.6	0.261		
	Yes	35.1			
Coronary artery disease	No	41.1	0.458		
	Yes	25.3			
Cerebral vascular event	No	38.9	0.694		
	Yes	20.1			
Chronic lung disease	No	37.2	0.964		
	Yes	25.5			
Essential hypertension	No	52.4	0.153		
	Yes	28.0		`	
Dyslipidemia	No	36.8	0.807		
	Yes	22.2			
Liver cirrhosis	No	35.7	0.787		
	Yes	50.0			
Arterial fibrillation	No	36.1	0.719		
	Yes	35.0			
Diabetes mellitus	No	40.0	0.212		
	Yes	28.9			
Dialysis	No	43.1	0.056		
	Yes	19.0			
Regular antiplatelet or anticoagulant use (n)	No	39.2	0.274		
	Yes	30.4			
*Preoperative factors*					
WBC count out of normal range^b^	No	46.2	0.099		
	Yes	28.6			
Positive band form of WBC	No	63.8	0.000	Ref.	0.301
	Yes	16.0		1.495 (0.698–3.205)	
NLR	< 23.8	38.2	0.592		
	≥ 23.8	30.3			
Platelet count	≥ 100,000/uL	40.0	0.027	Ref.	0.055
	< 100,000/uL	26.9		2.189 (0.983–4.874)	
INR of PT	≤ 1.7	40.6	0.000	Ref.	0.012
	> 1.7	9.1		3.006 (1.278–7.071)	
Hemoglobin	≥ 12 g/dL	46.8	0.151		
	< 12 g/dL	28.9			
Serum albumin	≥ 2.8 g/dL	45.5	0.008	Ref.	0.337
	< 2.8 g/dL	24.2		1.359 (0.727–2.541)	
Serum ALT	< 100 U/L	39.6	0.002	Ref.	0.858
	≥ 100 U/L	22.2		0.917 (0.354–2.377)	
Serum bilirubin	< 2.0 mg/dL	39.6	0.049	Ref.	0.437
	≥ 2.0 mg/dL	23.7		0.319 (0.656–2.655)	
Serum creatinine	< 2.0 mg/dL	52.5	0.017	Ref.	0.413
	≥ 2.0 mg/dL	28.6		1.409 (0.620–3.201)	
Shock status	No	44.9	0.000	Ref.	0.731
	Yes	18.6		1.149 (0.520–2.541)	
*Surgical factors*					
Time to surgery^c^	< 12 h	38.7	0.903		
	≥ 12 h	28.7			
Intraoperative red blood transfusion	No	35.3	0.873		
	Yes	38.9			
Combined colon resection	No	42.7	0.027	Ref.	0.264
	Yes	19.2		1.605 (0.700–3.677)	
End-enterostomy	No	58.8	0.006	Ref.	0.070
	Yes	24.5		2.525 (0.926–6.880)	
Functional residual small intestine	≥ 100 cm	45.3	0.019	Ref.	0.802
	< 100 cm	20.4		1.098 (0.528–2.281)	
*Postoperative medical factor*					
Oral antiplatelets	No	25.5	0.001	Ref.	0.007
	Yes	61.7		0.281 (0.112–0.704)	

*WBC* white blood cell; *NLR* neutrophil-to-lymphocyte ratio; *INR* international normalized ratio; *PT* prothrombin time; *ALT* alanine aminotransferase; *CI* confidence interval; *Ref.* reference

^a^Experiencing major cardiovascular procedure within 90 days before intestine resection

^b^Normal range of WBC: between 4000 and 12,000/uL

^c^Period between definite diagnosis by contrasted computed tomography and surgery

## Discussion

Intestinal gangrene is a severe event with a poor prognosis in patients with AMI [5]. Early diagnosis and intervention, especially within 12 h, can reduce mortality and preserve a more viable intestine [18, 19]. However, when intestinal gangrene and peritonitis develop, immediate laparotomy must be performed to achieve curative treatment [20, 21]. Enteral medication is always unavailable in the short critical period after intestinal resection. LMWHs are suggested in all patients undergoing revascularization and/or intestine resection during the critical postoperative period [13]. In the current study, patients with postoperative parenteral anticoagulants, especially enoxaparin, had a lower mortality rate after intestinal resection, despite the absence of revascularization. It also showed a benefit on survival.

LMWHs have more effective anti-Xa activity than anti-IIa activity, a more predictable anticoagulant response, and a lower incidence of heparin induced thrombocytopenia [22]. Enoxaparin also decreases platelet activity through COX1 in patients with coronary artery disease [23]. Clinically, enoxaparin can be used in patients with atrial fibrillation, pulmonary embolism, and deep vein thrombosis due to its antithrombotic activity [24, 25]. Enoxaparin can also be used in patients with unstable angina and non-ST-elevation myocardial infarction during the period of percutaneous coronary intervention [26]. The main effect of enoxaparin is its direct antithrombotic activity, as well as indirect antiplatelet activity.

In patients with sepsis, hypercoagulation is induced by impairing both the protein C and antithrombin systems [27]. Inappropriate accumulation and activity of platelets contribute to hyperinflammation and microthrombosis [28]. Severe sepsis, classically associated with gram negative bacterial infection, eventually leads to disseminated intravascular coagulation and multiple organ dysfunction syndrome [29]. In patients with mesenteric ischemia, both platelet-endothelial cell interactions and platelet-leukocyte adhesion also contribute to inflammation during intestinal ischemia/reperfusion injury [30]. LMWHs may treat or prevent severe hypercoagulation in patients with sepsis [31, 32].

During the period of intensive care, anticoagulant therapy with heparin should be initiated to prevent the progression of the thromboembolic occlusive process within the intestinal arteries [33]. Heparinization is routinely administered while patients underwent vascular procedures [19, 34]. Long-term use of anticoagulants (warfarin or low-molecular weight heparin) has also provided advantages [35]. In the present study, all patients had intestinal gangrene and underwent treatment by resection. This implies that all patients may have had intestinal microorganism overgrowth, although not all patients had positive bacterial cultures from ascites or blood. Sixty-two patients (72.9%) had abnormal WBC counts or immature WBCs before surgery (data not shown). Seventy-four patients (87.1%) had 2 or more points on the sepsis-related organ failure score (SOFA score [36], data not shown). Most of our patients experienced both AMI and sepsis. Postoperative anticoagulants may prevent recurrent vascular thrombosis/embolism, sepsis-induced hypercoagulation, and inappropriate platelet activity inducing ischemia/reperfusion intestinal injury.

After a short period of critical illness, enteral antiplatelet or anticoagulant therapy is suggested to avoid recurrent ischemia events. Patients with hypertension or diabetes are at high risk of coronary thrombosis and should be treated by antiplatelet therapy [2]. Patients with atrial fibrillation, accounting for one-third of EAMI cases, should be treated using an anticoagulant [37, 38]. In our study, sixty-one patients (72%) and 34 patients (40%) had essential hypertension and type 2 diabetes, respectively. Twelve patients had atrial fibrillation (14%). Thirty-four patients (60.7%) in Group B had enteral antiplatelet or anticoagulant treatment after surgery. The antiplatelets drugs included clopidogrel and aspirin, and the anticoagulants included warfarin and rivaroxaban.

The risk factors for mortality include advanced age (more than 70 years), diabetes mellitus, use of digoxin, delayed surgery, shock status, metabolic acidosis, hypoalbuminemia, recent myocardial infarction, previous cardiac surgery, and colonic involvement [9, 11, 39, 40]. In-hospital mortality, 30-day mortality, and 90-day mortality were used for analysis in previous studies. In-hospital mortality is too erratic to represent an accurate postoperative interval. For example, a patient who survived at first admission and died within 30 days of the postoperative period would be categorized into the survival group. To elucidate an accurate prognosis, we presented 30-day mortality for short-term results and survival curves for long-term results. In the present study, patients treated with postoperative parenteral anticoagulants showed a lower 30-day mortality rate and a better 2-year survival rate.

The control of confounding factors is difficult because these patients underwent critical emergent surgery, and had highly different medical histories and preoperative conditions. The patient number is also small because of the low incidence in the eastern world. A major limitation in this study was the dosage of anticoagulant. Due to the retrospective method employed in this study, dosage control was not achievable. The doses chosen by the physician might have been influenced by the patients’ serum creatinine levels, blood platelet counts, INR of PT, and perioperative bleeding events. Two patients with postoperative severe bleeding events, namely, intracranial hemorrhage and gastrointestinal bleeding, were in Group A. Another study limitation involved the periods of parenteral anticoagulant administration and the shifting to enteral antiplatelet/anticoagulant therapy. The period of enoxaparin use was between 1 and 32 days. In Group B, 8 patients had a short period of use (less than 3 days), and in 5 of these cases, this brevity was due to mortality within 3 days after surgery. It was not possible to reach a definite conclusion regarding the optimal beneficial dosage and period based on our research, and thus further study is necessary to shed light on this issue.

The appropriate clinical scenario may indicate that patients experience a critical period with unavailable enteral medication after emergent small bowel surgery. During this period, parenteral anticoagulants can be used for bridging treatment. While bowel movement redevelops, oral antiplatelet treatments can be initiated for long-term use.

## Conclusions

Immediate postoperative parenteral anticoagulant use can increase survival in patients with *AMI* and intestinal gangrene after therapeutic resection. In our experience, subcutaneous enoxaparin is a feasible choice for such patients during the critical postoperative period.

## Data Availability

The data generated and analyzed during the current study are not publicly available because the Taiwan (R.O.C.) government legally restricts by Personal Data Protection Act and Human Subjects Research Act. Data however are available from the corresponding author with de-linkage and de-identification on reasonable request.

## Abbreviations

AMI: Acute mesenteric ischemia
EAMI: Arterial embolism
TAMI: Arterial thrombosis
NOMI: Non-occlusive mesenteric ischemia
VAMI: Venous thrombosis
LMWHs: Low-molecular-weight heparins
CT: Computed tomography
NLR: Neutrophil-to-lymphocyte ratio
ALT: Alanine aminotransferase
PT: Prothrombin time
INR: International normalized ratio
